# Radio Frequency Nonionizing Radiation in a Community Exposed to Radio and Television Broadcasting

**DOI:** 10.1289/ehp.8237

**Published:** 2005-09-20

**Authors:** James B. Burch, Maggie Clark, Michael G. Yost, Cole T.E. Fitzpatrick, Annette M. Bachand, Jaya Ramaprasad, John S. Reif

**Affiliations:** 1 Department of Epidemiology and Biostatistics, University of South Carolina, Columbia, South Carolina, USA; 2 Department of Environmental and Radiological Health Sciences, Colorado State University, Fort Collins, Colorado, USA; 3 Department of Environmental and Occupational Health Sciences, University of Washington, Seattle, Washington, USA

**Keywords:** broadcasting, electromagnetic fields, exposure assessment, GIS, nonionizing radiation, radio, television

## Abstract

Exposure to radio frequency (RF) nonionizing radiation from telecommunications is pervasive in modern society. Elevated disease risks have been observed in some populations exposed to radio and television transmissions, although findings are inconsistent. This study quantified RF exposures among 280 residents living near the broadcasting transmitters for Denver, Colorado. RF power densities outside and inside each residence were obtained, and a global positioning system (GPS) identified geographic coordinates and elevations. A viewshed model within a geographic information system (GIS) characterized the average distance and percentage of transmitters visible from each residence. Data were collected at the beginning and end of a 2.5-day period, and some measurements were repeated 8–29 months later. RF levels logged at 1-min intervals for 2.5 days varied considerably among some homes and were quite similar among others. The greatest differences appeared among homes within 1 km of the transmitters. Overall, there were no differences in mean residential RF levels compared over 2.5 days. However, after a 1- to 2-year follow-up, only 25% of exterior and 38% of interior RF measurements were unchanged. Increasing proximity, elevation, and line-of-sight visibility were each associated with elevated RF exposures. At average distances from > 1–3 km, exterior RF measurements were 13–30 times greater among homes that had > 50% of the transmitters visible compared with homes with ≤ 50% visibility at those distances. This study demonstrated that both spatial and temporal factors contribute to residential RF exposure and that GPS/GIS technologies can improve RF exposure assessment and reduce exposure misclassification.

Public exposure to radio frequency (RF) fields from sources such as radio and television (TV) broadcasting is common in industrialized countries. As of 1999, an estimated 14,000 radio and TV stations were on the air in the United States [Federal Communications Commission (FCC) 1999]. Public concerns about the potential health effects of exposure to RF non-ionizing radiation stem partly from the pervasiveness of these exposures. The primary toxicologic response associated with RF exposure occurs through tissue heating, and current exposure standards for the general public are based primarily on a thermal mechanism of action [FCC 1999; National Radiological Protection Board (NRPB) 2003]. Ambient RF power densities from telecommunications devices are typically well below these levels (NRPB 2003). However, the issue of whether health effects occur at nonthermal RF exposure levels in the general population remains controversial. Elevated cancer rates have been reported in some residential human populations exposed to radio and TV transmissions (Ahlbom et al. 2004). Nonthermal RF exposures may also be linked with hematologic, neurologic, reproductive, and cardiovascular disorders [NRPB 2003; World Health Organization (WHO) 1993]. Results from studies published to date have not established a clear cause–effect relationship between RF exposure from radio and TV broadcasting and adverse health outcomes in humans. Previous epidemiologic investigations of the relationship between residential RF exposure and human cancers have relied almost exclusively on distance to RF transmitters to characterize exposure (Ahlbom et al. 2004). Few studies have examined factors that contribute to spatial or temporal variation in residential RF exposure (Allen 1991; Anglesio et al. 2001; Dahme 1999; Mantiply et al. 1997; Tell and Mantiply 1980). Potential limitations with respect to exposure misclassification may therefore apply to previous studies of residential RF exposures and adverse health effects.

Populations residing near telecommunications broadcasting installations tend to have the highest nonoccupational RF exposures. Lookout Mountain in Golden, Colorado, is a residential community that contains numerous radio and TV transmitters that broadcast to the entire Denver metropolitan area. The topography of Lookout Mountain may place some homes within a main beam of these transmissions [U.S. Environmental Protection Agency (EPA) 1987]. Several publicly accessible areas closest to these towers have exceeded the general public nonionizing radiation standard (200 μW/cm^2^) on each occasion that they were measured (Cleveland 1998; Jefferson County Department of Health and Environment 1996; U.S. EPA 1987). The Colorado Department of Public Health and Environment (2004) recently reported a statistically significant increase in brain cancer rates among residents in two census block groups in close proximity to the transmitters on Lookout Mountain. Although the limited number of cases available for study and a lack of detailed exposure assessment limit the interpretation, these findings indicate a need for further research in this community. The purpose of this investigation was to develop a better understanding of the temporal and spatial characteristics of residential RF exposures from radio and TV broadcasting by studying the Lookout Mountain community. We evaluated temporal characteristics of RF exposure by comparing repeated measurements collected over several different time scales. We determined spatial characteristics of RF exposure using spot measurements within and outside homes. We evaluated broader spatial variability using a geographic information system (GIS) that combined spot RF measurements with geographic parameters (distance, elevation, and line-of-sight visibility from the broadcast antennae).

## Materials and Methods

**Figure 1:**
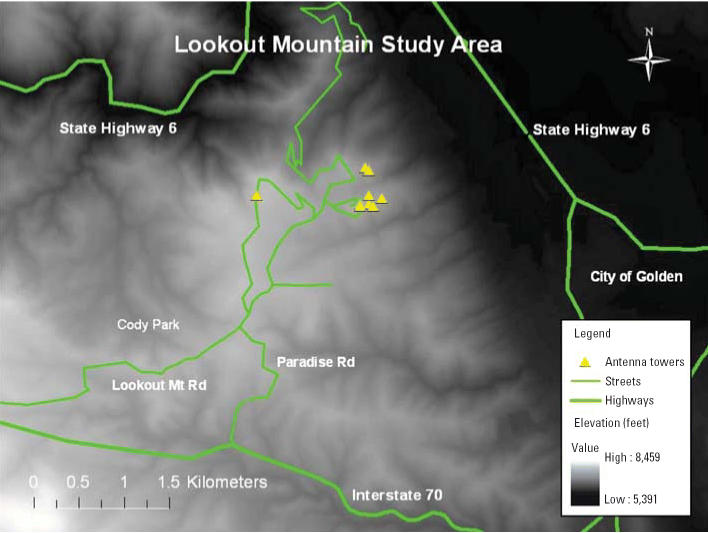
Map of the study area, Lookout Mountain, Golden, Colorado (only major highways and primary roads are shown).

The study area was defined by Interstate 70 (I-70) to the south, the Cody Park neighborhood to the west, and the natural topography that drops steeply in elevation to the north and east of the Lookout Mountain community (Figure 1). No homes < 7,100 ft in elevation were included in the study area. The source population was identified by matching Jefferson County Zoning Office files to the U.S. Postal Service’s rural route information, which yielded 94% concordance among the residences identified. A population census of each person living in these residences ≥ 8 years of age identified approximately 576 homes and 1,375 individuals in the study area. After a curbside RF survey along publicly accessible streets, a sample of 441 eligible study participants (≥ 8 years of age ) was identified for recruitment among strata of high (> 4.0 μW/cm^2^), medium (0.5–4.0 μW/cm^2^), and low (< 0.5 μW/cm^2^) RF exposure categories. All adults from the high-RF–exposure category and all children (8–18 years of age) from the high and medium categories were considered eligible. Individuals in the remaining categories were randomly selected from the census database.

Spatial averages of RF field intensity were obtained at one exterior and five interior locations (bedroom, kitchen, living room, computer room or office, room most used) at each residence using a Narda EMR-300 meter with a type 18 isotropic E-field probe (flat response, 0.1–3,000 MHz; detection limit, 0.2 V/m; Narda Safety Test Solutions, Pfullingen, Germany). In each room, a spatial average that included the approximate room center and four points midway between the room center and each corner was recorded at approximately 1 m above the floor. RF fields were also measured at computer work stations directly in front of the monitor where the operator was typically seated, while the computer was operating. Exterior RF measurements were made on the side of the house closest to the transmitters, usually on the yard, patio, or driveway. Measurements were made at the beginning of a 2.5-day data collection period (typically Friday afternoon or evening) and repeated at the end of this period (usually Monday morning). Information on the presence of RF shielding and wireless Internet service and the number of cordless and cellular telephones used in the home was recorded. Data were collected weekly from September 2002 through December 2003 among four to eight participants with a range of RF exposures. In a subset of 17 homes, RF power densities were logged continuously at 1-min intervals during the 2.5 day data collection period using the RF meter adapted to a personal computer for data storage. Spot measurements were also repeated in a subset of homes 8–29 months later at 8 interior and 12 exterior locations.

Radio and TV transmitters in this area emit approximately 9 MW of broadcast power and are deployed in three groups of antenna towers (northeast, northwest, and southeast) located approximately 0.4–1.2 km apart (Figure 1). Geographic and telecommunications characteristics of 15 primary transmitters in this area are presented in Table 1 (FCC 2004). Residences in the study area are located south and southwest of the RF transmitters identified in Figure 1. The only mobile telephone tower identified in this area was located within 100 m of I-70 in the southwest corner of the study area; the nearest participant lived approximately 2 km to the northeast, where RF spot measurements were typically below the limit of detection. A global positioning system (GPS) was used to identify the geographic coordinates and elevation of each exterior RF measurement (eTrex Legend, part 190-00234-00; Garmin International Inc., Olathe, KS). The distance between each transmitter and residence was characterized using the ESRI ArcInfo program (ESRI, Redlands, CA). A command line program was written in Arc Macro Language that computed the distance between a set of fixed points (transmitters) and input points (residential GPS coordinates), and the results were compiled in a Microsoft Excel spreadsheet. Line-of-sight visibility was characterized using the viewshed analysis tool of ESRI ArcMap 9.0 as part of a GIS. The view-shed model determined line-of-sight visibility (yes/no) using a set of input points (transmitters) and a three-dimensional input surface (digital elevation model). The viewshed results were overlaid on a GIS of points (residential coordinates) and the results tabulated. The average distance from each residence to the 15 transmitters identified in Table 1 and the percentage of these transmitters visible from the home were used in the final analysis.

Statistical analyses were performed using the SAS computer program (version 9.1; SAS Institute Inc., Cary, NC). Exterior and interior RF measurements were converted to micro-watts per square centimeter because residential RF measurements obtained in this study were expected to be in the far field (Dahme 1999). Values below the limit of detection were given a value of one-half the detection limit (0.01 μW/cm^2^) for analysis. Analyses were performed using log-transformed RF values, and results were converted back to microwatts per square centimeter for presentation. Attenuation of RF levels from the exterior to the interior of each home was calculated as the difference between the exterior value and the interior house average, divided by the exterior value, and represented as a percentage. Spearman correlation coefficients (*r*) were used to evaluate relationships among RF measurements between rooms and among days. The SAS Proc Mixed procedure for repeated measures computed maximum likelihood estimates of factors potentially influencing RF values as fixed effects (shielding, distance, elevation, percentage of transmitters visible) along with “day” in the statistical model using an unstructured covariance matrix. Initially, residences were grouped into quartiles by distance, elevation, or visibility, and least-squares means of exterior or interior house average RF power densities were calculated within each quartile. We compared mean RF levels in the lowest and highest quartiles using the least significant differences statistic in SAS. Similar analyses were used to evaluate other categorical variables (attenuation, shielding) as well as combined effects (e.g., distance and visibility). Multivariate analyses included each predictor simultaneously in the statistical model and the statistical significance of each effect was evaluated based on type III sums of squares. Results presented below were not altered when the locations of three TV transmitters on Mount Morrison (5 MW each, ~ 2.5 km south of I-70) were included in the analysis. Results were unchanged when analyses were restricted to only one subject per home, except in one case, noted below. Adjustment for RF shielding in the home did not alter the results for interior house average power densities.

## Results

We obtained 560 RF spot measurements for 280 randomly selected residents living in 161 different homes. The study participation rate was 64%, and mean age and RF power densities did not differ among participants and nonparticipants (data not shown). Exterior RF values ranged from nondetectable to 20.9 μW/cm^2^ (mean ± SD = 2.6 ± 4.0 μW/cm^2^), all below the general public exposure limit (200 μW/cm^2^). House average power densities ranged from nondetectable to 6.7 μW/cm^2^ (mean ± SD = 0.8 ± 1.0 μW/cm^2^).

### Temporal RF exposure characteristics.

**Figure 2:**
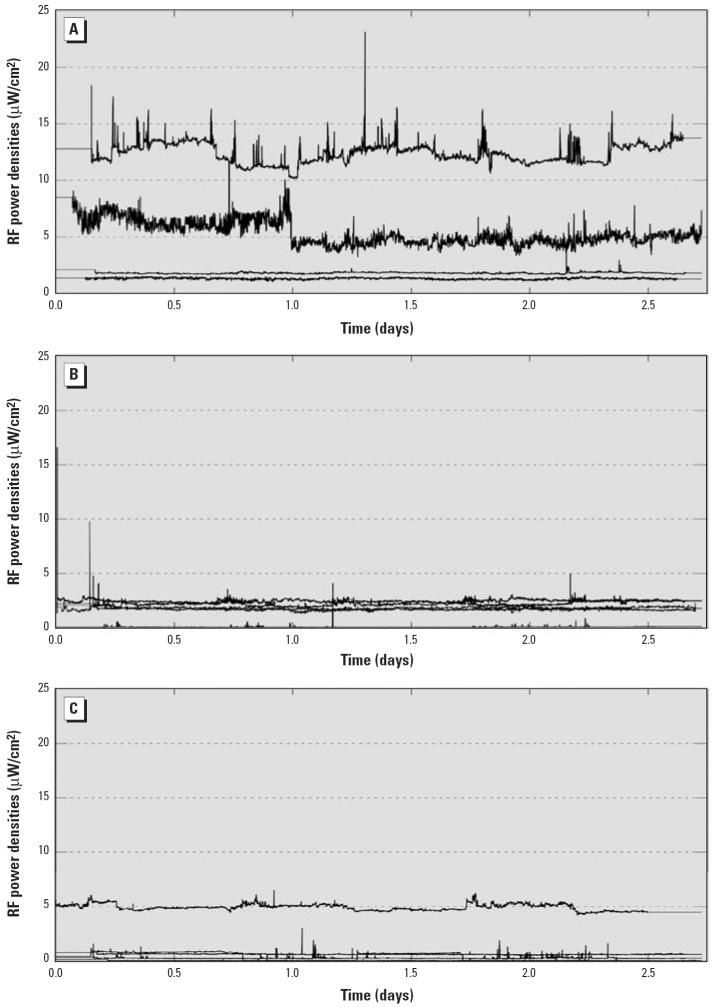
RF power densities recorded at 1-min intervals for homes ≤ 1 km (*A*), > 1–2 km (*B*), or > 2–3 km (*C*) from the transmitters. Each time series was collected from a stationary location within a single home on a different day. Results have been superimposed so that the time of day coincides among traces. Time 0.0 corresponds to 13:40. A flat line designates times before or after the recording period (i.e., no data collection).

The temporal variability in RF exposure within and among homes is illustrated in Figure 2. Time series of 1-min RF measurements collected among 14 homes within 1 km (*n* = 4), between 1 and 2 km (*n* = 5), and from 2 to 3 km (*n* = 5) mean distance from the transmitters are presented. Each time series begins at the same time of day (13:40) but was collected on different days (Figure 2). Two traces in the 1–2 km group and one within the 2–3 km group were excluded because the data could not be easily distinguished from another trace.

Residential RF spot measurements taken at the same location approximately 2.5 days apart were well correlated (exterior RF measurements *r* = 0.99, *p* < 0.001; interior house average *r* = 0.97, *p* < 0.001). We found no statistically significant differences in mean exterior or interior power densities between days, either using all data combined or comparing days within categories of distance from the transmitters (≤ 1, > 1–2, > 2–3, or > 3 km; data not shown). When the analysis was restricted to one subject per home, there was a statistically significant difference between days for exterior average RF levels at distances > 2–3 km from the transmitters, although the absolute difference was small (0.10 vs. 0.14 μW/cm^2^, *p* ≤ 0.01), and no other differences between days were noted for mean exterior or interior RF levels.

After a mean (± SD) follow-up of 14 ± 3 months, average RF measurements within the same homes were 12 ± 20% lower (38% did not change). The average absolute difference between interior house average readings taken at the beginning and end of the 14 ± 3 month follow-up period was –0.09 ± 0.14 μW/cm^2^. Exterior RF measurements collected at the same locations 17 ± 6 months apart increased by 15 ± 72% (25% did not change, 25% increased by > 100%). The average absolute difference in exterior RF measurements was 1.1 ± 1.9 μW/cm^2^ over that follow-up period.

### Spatial RF exposure characteristics.

Among homes with detectable RF, the average attenuation from outside to inside the home was 44 ± 62%; 12% of subjects had higher RF levels inside their home, and 5% had ≤ 10% RF attenuation from exterior to interior. Among subjects reporting the installation of RF shielding on windows or other locations within the home (*n* = 29), residential RF power densities were attenuated an average of 64% compared with 39% among those without RF shielding (*n* = 225, *p* = 0.05). There was a strong positive correlation among RF measurements from different rooms in the home (*r* ≥ 0.73). Maximum RF values in homes were most strongly correlated with measurements in the living room (*r* = 0.93) and bedroom (*r* = 0.92). Comparisons of RF levels resulting from home computers and wireless Internet were made at the farthest distances from the transmitters to avoid interference from radio and TV broadcasting. At distances > 3 km from the transmitters, the average power density at computer work stations was 0.15 ± 0.30 μW/cm^2^ (*n* = 21). The house average power densities among homes with and without wireless Internet service were 0.31 ± 0.22 μW/cm^2^ (*n* = 3) and 0.09 ± 0.31 μW/cm^2^ (*n* = 73, *p* = 0.06), respectively, at distances > 2–3 km from the broadcast antennae. No participants living > 3 km reported wireless Internet use.

The elevation of homes in this study ranged from 7,123 to 7,782 feet above sea level (interquartile range, 7,332–7,499 feet; mean ± SD, 7,408 ± 117 feet). One-quarter of the study population lived at an elevation above five of the transmitters listed in Table 1 [27% of those within 1 km (*n* = 19), 46% of subjects from > 1 to 2 km (*n* = 32), and 19% of those > 2 km (*n* = 13)]. The average distance of each home to the 15 transmitters in Table 1 ranged from 0.4 to 5.7 km (mean ± SD = 1.7 ± 0.9 km). House average power densities are presented in Table 2 by quartiles of distance (≤ 1, 1.1–1.5, 1.6–2.3, > 2.3 km), elevation (≤ 7,332, 7,333–7,399, 7,400–7,499, > 7,499 feet), and line of sight (≤ 16.7%, 16.8–53.3%, 53.4–80.0%, > 80% of transmitters visible). Increasing proximity, elevation, and visibility were each associated with statistically significant increases in mean exterior and interior RF power densities (*p* ≤ 0.01; Table 2).

Table 3 presents average interior and exterior residential power densities stratified by distance to towers (≤ 1, > 1–2, > 2–3, or > 3 km) and by percentage of towers visible at each residence (≤ 50% or > 50%). In both strata of visibility, there was a clear gradient of increasing mean power densities within and outside homes with increasing proximity to the transmitters. Homes with > 50% of transmitters visible by viewshed analysis that were also within 1 km of the antennae had mean exterior RF levels that were approximately 2 times greater and average interior levels that were 1.4 times greater than homes with ≤ 50% of transmitters visible at the same distance. However, these differences were not statistically significant (*p* = 0.10 and 0.26, respectively). At average distances of > 1–2 km or > 2–3 km with > 50% of transmitters visible, exterior RF measurements were approximately 13–30 times greater and interior RF power densities were 4 to 8 times greater than those in homes with ≤ 50% visibility (all *p* ≤ 0.001; Table 3). Interior average power densities > 3 km from the antennae were not elevated among homes with ≥ 50% of the transmitters visible, and many readings were close to or at the limit of detection. However, exterior RF levels at that distance were approximately 3 times greater among homes with > 50% of transmitters visible (*p* = 0.05; Table 3).

Table 4 presents average interior and exterior residential power densities stratified by distance to the towers and by quartile of elevation at each residence. Increasing residential elevation contributed to increases in mean exterior or interior RF exposures only at certain distances from the towers, primarily between 1 and 3 km (Table 4). There was a weak positive correlation between elevation and both distance (r = 0.12) and percentage of transmitters visible (r = 0.15), and a modest negative correlation between distance and percentage of transmitters visible (r = -0.55). When each of these factors was incorporated as a continuous variable into a multivariate statistical model, increased proximity and transmitter visibility were each associated with statistically significant increases in interior house average RF power densities (both p < 0.01), whereas elevation was not (p = 0.10). However, increased proximity, percentage of transmitters visible, and elevation were all independently associated with increased exterior RF power densities (all p < 0.01) (data not shown).

## Discussion

As the debate regarding the health implications of exposure to nonionizing radiation continues, an increased effort must be made to evaluate factors that predict exposure to RF fields. To our knowledge, this was the first study to examine both the temporal and spatial characteristics of residential RF exposure to broadcast antennae. In 1980, the U.S. EPA surveyed background RF radiation among 15 cities across the United States (Tell and Mantiply 1980). Median RF exposure to the general public was estimated at 0.005 μW/cm^2^, and about 99% of public exposure was estimated to be < 1 μW/cm^2^ (Tell and Mantiply 1980). Although none of the residential power densities in the present study exceeded the general public exposure limit of 200 μW/cm^2^ (NRPB 2003), approximately one in four Lookout Mountain participants had average RF levels > 1 μW/cm^2^ inside their homes.

The accurate characterization of an individual’s exposure over extended time periods is one of the most difficult problems to overcome when investigating the chronic health effects of environmental agents in human populations. In this study, we evaluated temporal variation in residential RF power densities on several scales ranging from minutes to years. Continuously recorded 1-min power density measurements were used to characterize short-term temporal variation of RF power densities within homes from radio and TV broadcasting. Within each stratum of 1-km distance, time series of power density measures varied considerably among some homes and were quite similar among others (Figure 2). The greatest differences appeared among homes closest to the transmitters, which suggests that changes in broadcasting patterns may contribute to RF exposure among some people living in close proximity to the transmitters. The results highlight the importance of characterizing exposures at the individual level and suggest that measurement timing may affect the accuracy of exposure classification.

When average residential RF levels were compared over several days, there were no differences in mean RF exposure at the same residences. However, results were less consistent when RF levels were compared over time scales ranging from 0.7 to 2.4 years. RF levels did not change in approximately 25–38% of the residences in which long-term comparisons were made, and the average absolute change in RF power densities was small (≤ 1 μW/cm^2^). However, there was considerable variation among repeated measurements, resulting in a 12% decrease in interior house average power densities but a 72% increase in exterior RF levels. In one case, a 176% increase in the exterior RF follow-up measurement could have been caused by the removal of a tree near the residence. In another case, a 50% reduction in the interior RF follow-up measurement may have been associated with the reported installation of RF shielding within the home between measurements. Differences in the time of day that repeat measurements were performed may have also contributed to the observed differences. If left uncharacterized, temporal variations in RF exposure could increase uncertainty and obscure the ability to evaluate the long-term effects of residential nonionizing radiation exposures. Interpretation of the results for temporal RF fluctuations in this study is limited by the subset of homes available for analysis. However, the results indicate that temporal RF exposure patterns are stable for some subjects but in other cases relatively large differences may exist within and among subjects over time.

In this study, an overall 44% reduction in RF levels from outside to inside homes was observed, and installation of RF shielding within the home decreased RF power densities by an additional 45%. In some cases however, interior RF levels were approximately the same or greater than exterior power densities (~ 17% of the subjects studied). This somewhat unexpected finding suggests that in some cases residential sources of RF exposure (e.g., computer monitors or wireless computer networks) may have contributed to interior RF levels to a greater extent than external radio and TV transmitters. For example, homes with wireless Internet service at distances > 2 km had average interior RF levels that were more than 3 times greater than homes without these devices. Minimal differences between interior and exterior measurements may have also been due to the increased elevation and lack of external obstructions outside the upper floors of some residences. An increase in RF levels on the upper floors of buildings has been reported previously (Anglesio et al. 2001). The strong correlation between maximum interior RF measurements and measurements in bedrooms and living rooms, which are likely to be located on upper floors, also supports this possibility. Scattering or reflection from local terrain, buildings, or vegetation may have also influenced indoor–outdoor differences.

Prior epidemiologic investigations of the relationship between residential RF exposures and various health outcomes have relied on distance from transmitters to assess exposure (Ahlbom et al. 2004; NRPB 2003; WHO 1993). Results from this study indicate that distance was a strong predictor of RF power density measurements on Lookout Mountain, but that line of sight and elevation also had an important impact on residential RF levels. One in four of the study subjects lived at elevations that were higher than several of the major transmitters in this area. Increasing elevation enhanced residential RF exposures, but this effect varied by distance, contributing most at distances between 1 and 3 km from the transmitters. The results support a previous assertion that the topography of Lookout Mountain may place some homes at elevations that are within the main beam of the RF transmissions (U.S. EPA 1987). Homes with > 50% line-of-sight visibility that were 1–3 km from the transmitters had RF levels that were much greater than those with ≤ 50% visibility at the same distance. The results are consistent with theoretical properties of radio wave propagation and confirm that line-of-sight visibility is an important independent predictor of RF exposure from broadcast transmitters. With the increasing use of RF technologies in modern society, including the installation of mobile telephone and wireless Internet transmitters in urban environments, ambient RF levels will continue to climb. Refined exposure assessment methodologies are critical for improving epidemiologic investigations of RF exposures (Ahlbom et al. 2004). This study demonstrated the feasibility of using GPS/GIS technologies to improve RF exposure assessment and reduce exposure mis-classification. Proximity, elevation, line of sight, alternate sources, and temporal variability each contributed to RF exposure and should be evaluated in future investigations of the potential health effects of RF broadcasting in human populations.

## Figures and Tables

**Table 1:** Characteristics of radio and television broadcast transmitters, Lookout Mountain, Golden, Colorado (March 2004).

Antenna group/frequency (MHz)	Elevation (feet above sea level)	Latitude (°)	Longitude (°)	ERP (kW)	Antenna azimuth pattern
Northwest					
83.25	7,707	39.73028	105.25000	100	O
Northeast					
55.25	7,684	39.73278	105.23556	100	O
105.90	7,520	39.73278	105.23556	96	O
106.70	7,520	39.73278	105.23556	96	O
95.70	7,412	39.73306	105.23611	100	D
687.25	7,376	39.73278	105.23556	2,510	D
Southeast					
67.25	8,078	39.73000	105.23389	100	O
573.25	7,701	39.72917	105.23667	5,000	D
175.25	7,664	39.72944	105.23667	316	O
90.10	7,655	39.73028	105.23556	45	O
89.30	7,655	39.73028	105.23556	22.5	O
187.25	7,609	39.72944	105.23556	316	O
99.50	7,422	39.72917	105.23500	74	D
101.10	7,422	39.72917	105.23500	74	D
103.50	7,028	39.73056	105.23528	100	O

Abbreviations: D, directional; ERP, effective radiated power; O, omnidirectional.

**Table 2:** Exterior and interior RF power densities (μW/cm^2^): effect of distance, elevation, and line of sight, Lookout Mountain, Golden, Colorado.

	Quartile				
	1	2	3	4	*p*-Value, 1 vs. 4
Exterior average					
Distance^a^	2.93	1.28	0.38	0.07	< 0.001
Elevation	0.42	0.55	0.32	1.42	< 0.001
Percent visible^b^	0.06	0.61	1.58	3.84	< 0.001
Interior house average					
Distance^a^	1.05	0.73	0.21	0.05	< 0.001
Elevation	0.26	0.37	0.17	0.51	0.010
Percent visible^b^	0.07	0.38	0.53	1.14	< 0.001

a Average distance to 15 major RF (see Table 1) transmitters in area.

b Percentage of transmitters visible from the residence.

**Table 3:** Exterior and interior RF power densities by distance and line of sight, Lookout Mountain, Golden, Colorado [μW/cm^2^ (no. of subjects)].

		Percent of transmitters visible		
Distance to transmitter	All subjects (*n* = 280)	≤ 50	> 50	*p*-Value, ≤ 50% vs. > 50%
Exterior average				
≤ 1 km	2.92 (62)	1.67 (11)	3.32 (51)	0.100
> 1–2 km	1.00 (117)	0.21 (46)	2.66 (71)	< 0.001
> 2–3 km	0.13 (76)	0.05 (54)	1.49 (22)	< 0.001
> 3 km	0.06 (25)	0.04 (18)	0.13 (7)	0.050
*p*-Value, ≤ 1 vs. > 3 km	< 0.001	< 0.001	< 0.001	
Interior house average				
≤ 1 km	1.14 (62)	0.86 (11)	1.22 (51)	0.260
> 1–2 km	0.53 (117)	0.22 (46)	0.92 (71)	< 0.001
> 2–3 km	0.09 (76)	0.05 (54)	0.39 (22)	< 0.001
> 3 km	0.03 (25)	0.04 (18)	0.01 (7)	0.020
*p*-Value, ≤ 1 vs. > 3 km	< 0.001	< 0.001	< 0.001	

**Table 4:** Exterior and interior RF power densities by distance and elevation, Lookout Mountain, Golden, Colorado [μW/cm^2^ (no. of subjects)].

	Elevation quartile				
Distance to transmitter	1	2	3	4	*p*-Value, 1 vs. 4
Exterior average					
≤ 1 km	2.79 (27)	2.76 (21)	4.54 (7)	3.10 (7)	0.87
> 1–2 km	0.59 (18)	0.84 (27)	0.86 (29)	1.51 (43)	0.03
> 2–3 km	0.04 (18)	0.07 (20)	0.12 (21)	1.37 (15)	< 0.01
> 3 km	0.04 (7)	0.01 (1)	0.05 (13)	0.32 (4)	0.03
*p*-Value, ≤ 1 vs. > 3 km	< 0.001	< 0.001	< 0.001	0.001	
Interior house average					
≤ 1 km	1.21 (27)	1.10 (21)	1.95 (7)	0.56 (7)	0.10
> 1–2 km	0.34 (18)	0.60 (27)	0.44 (29)	0.67 (43)	0.03
> 2–3 km	0.05 (18)	0.07 (20)	0.07 (21)	0.34 (15)	< 0.01
> 3 km	0.03 (7)	0.03 (1)	0.03 (13)	0.06 (4)	0.32
*p*-Value, ≤ 1 vs. > 3 km	< 0.001	< 0.001	< 0.001	< 0.001	
